# Elastography in the evaluation of thyroid nodules in children - expanding the field of ultrasound to reduce the need for invasive procedures

**DOI:** 10.1590/0100-3984.2019.52.3e1

**Published:** 2019

**Authors:** Silvia Maria Sucena da Rocha

**Affiliations:** 1 Attending Radiologist in the Department of Diagnostic and Therapeutic Support at the Instituto da Criança do Hospital das Clínicas da Faculdade de Medicina da Universidade de São Paulo (ICr/HC-FMUSP), Radiologist at the Laboratório Fleury Medicina e Saúde, São Paulo, SP, Brazil. E-mail: smsucena@gmail.com.

Thyroid nodules in children are always a cause for concern, because, although such nodules are rare, the risk of malignancy is high. Their management is therefore challenging. Children must be protected from unnecessary invasive procedures, such as fine-needle aspiration biopsy (for cytological study) and thyroidectomy. Nevertheless, in doing so, we risk missing the window for early treatment, thus allowing a malignant lesion to progress. Accordingly, the article by Cunha et al. "Elastography for the evaluation of thyroid nodules in pediatric patients"^(^^1^^)^, published in this issue of **Radiologia Brasileira**, addresses the contribution of ultrasound elastography to the differentiation between benign and malignant thyroid nodules in pediatric patients, based on the mechanical (elastic) properties of the lesions, and adds to the already well-established criteria for the detection of suspicious nodules on conventional and color Doppler ultrasound.

Ultrasound elastography is a new technique that, in a manner analogous to that of palpation, assesses tissue elasticity based on the principle that healthy tissues and benign lesions are usually less rigid than are tissues affected by malignant diseases^(^^2^^)^. The difference is that ultrasound elastography can express the consistency of an organ or lesion as a numerical value or a graphic representation. This promising technique has been employed in the evaluation of various organs and structures, such as the breast, thyroid gland, salivary glands, liver, spleen, kidneys, bowel loops, prostate, tendons, and muscles. Studies in this field are multiplying, and the technique has increasingly become more widespread and incorporated into the routine of examinations, because it has been credited with increasing the diagnostic accuracy of ultrasound, especially in the differentiation between benign and malignant thyroid nodules^(^^3^^)^, as well as in the identification of rare conditions such as intrathyroidal ectopic thymus^(^^4^^)^.

In diagnostic ultrasound, the limitations of the method that restricts the scope of the investigation must be taken into account. Nevertheless, it is potentially the ideal imaging method for evaluating children, because of its unique characteristics, including the ability to acquire multiplanar images, in real time, as well as because it is innocuous and painless. Therefore, it is with great enthusiasm that we, especially those of us who are pediatric radiologists, welcome any technological development that enhances and expands the scope of ultrasound.

We hope for more studies like this, the focus of this review, that will soon provide us with consensus parameters for the application of the technique. And we ask ourselves: will we evolve to the point of dispensing with biopsies?
